# Potential Impact of Antiretroviral Chemoprophylaxis on HIV-1 Transmission in Resource-Limited Settings

**DOI:** 10.1371/journal.pone.0000875

**Published:** 2007-09-19

**Authors:** Ume L. Abbas, Roy M. Anderson, John W. Mellors

**Affiliations:** 1 Division of Infectious Diseases, School of Medicine, University of Pittsburgh, Pittsburgh, Pennsylvania, United States of America; 2 Department of Infectious Disease Epidemiology, Imperial College, Faculty of Medicine, University of London, London, United Kingdom; McGill University AIDS Centre, Canada

## Abstract

**Background:**

The potential impact of pre-exposure chemoprophylaxis (PrEP) on heterosexual transmission of HIV-1 infection in resource-limited settings is uncertain.

**Methodology/Principle Findings:**

A deterministic mathematical model was used to simulate the effects of antiretroviral PrEP on an HIV-1 epidemic in sub-Saharan Africa under different scenarios (optimistic, neutral and pessimistic) both with and without sexual disinhibition. Sensitivity analyses were used to evaluate the effect of uncertainty in input parameters on model output and included calculation of partial rank correlations and standardized rank regressions. In the scenario without sexual disinhibition after PrEP initiation, key parameters influencing infections prevented were effectiveness of PrEP (partial rank correlation coefficient (PRCC) = 0.94), PrEP discontinuation rate (PRCC = −0.94), level of coverage (PRCC = 0.92), and time to achieve target coverage (PRCC = −0.82). In the scenario with sexual disinhibition, PrEP effectiveness and the extent of sexual disinhibition had the greatest impact on prevention. An optimistic scenario of PrEP with 90% effectiveness and 75% coverage of the general population predicted a 74% decline in cumulative HIV-1 infections after 10 years, and a 28.8% decline with PrEP targeted to the highest risk groups (16% of the population). Even with a 100% increase in at-risk behavior from sexual disinhibition, a beneficial effect (23.4%–62.7% decrease in infections) was seen with 90% effective PrEP across a broad range of coverage (25%–75%). Similar disinhibition led to a rise in infections with lower effectiveness of PrEP (≤50%).

**Conclusions/Significance:**

Mathematical modeling supports the potential public health benefit of PrEP. Approximately 2.7 to 3.2 million new HIV-1 infections could be averted in southern sub-Saharan Africa over 10 years by targeting PrEP (having 90% effectiveness) to those at highest behavioral risk and by preventing sexual disinhibition. This benefit could be lost, however, by sexual disinhibition and by high PrEP discontinuation, especially with lower PrEP effectiveness (≤50%).

## Introduction

While the search is ongoing for a safe and effective HIV-1 vaccine, encouraging data from animal studies [1]–[5] have ignited interest in pre-exposure chemoprophylaxis (PrEP) with antiretrovirals as a strategy to prevent HIV-1 infection [6]. The potential impact of targeted or widespread PrEP on HIV-1 epidemics is uncertain and major determinants of its utility have not been defined. Several clinical trials to address the efficacy of PrEP are underway, but these will take considerable time to complete and will not specifically address the potential public health benefit [7], [8]. We therefore developed a mathematical model of a heterosexual HIV-1 epidemic and analyzed the potential for HIV-1 prevention from PrEP under different scenarios of effectiveness, duration of use, population coverage, emergence and spread of drug resistance and increased sexual risk behavior.

## Methods

### Model Structure

**Figure 1:**
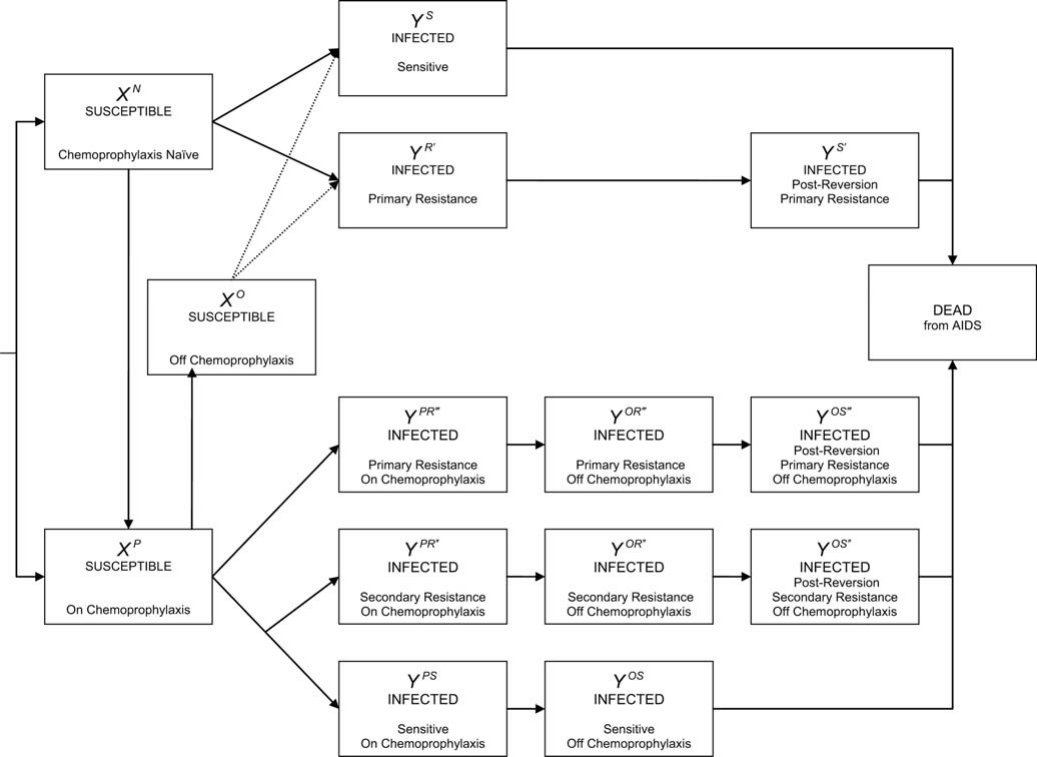
Simplified Flow Diagram of Model with PrEP Implementation.

We refined our previously described deterministic mathematical model of HIV-1 disease progression and heterosexual transmission by incorporating demographic and sexual behavioral details, and by the introduction of PrEP [9]. Briefly, the model population was stratified according to gender, age, sexual activity level, disease state, PrEP status, and HIV-1 drug resistance. Model input parameters were chosen to simulate a mature epidemic in southern sub-Saharan Africa. Parameter assignments were made from recent literature on HIV-1 disease progression [10], [11], infectivity [12], and sexual behavior [13]–[15]. The model consists of coupled nonlinear differential equations describing the population and epidemiological stratifications outlined in Figure 1. Model parameters are shown in Tables 1 and 2, and model equations and details are provided in the Appendix S1.

**Table 1:** Model Parameters for the Simulated HIV-1 Epidemic

PARAMETER	SYMBOL	VALUE	UNIT	REFERENCE
**Epidemiological**				
***Average Duration of HIV-1 Disease by Stage of Infection*** *				
Recent	*1/ω^1^*	0.5	year	[12]
Chronic	*1/ω^2^*	7.5	year	[10], [11]
AIDS	*1/ω^3^*	2.0	year	[10], [11]
***HIV-1 Infectivity by Stage of Infection*** *				
Recent	*γ^1^*	0.0082	per act	[12]
Chronic	*γ^2^*	0.0010	per act	[12]
AIDS	*γ^3^*	0.0036	per act	[12]
**Behavioral**				
Average rate of sexual partner change	*c*	2	per year	[13], [15]
Average number of sex acts per partnership for sexual activity levels 1 to 4Υ	*Ψ*	9, 23, 44, 120	per partner per year	[12], [53]
Assortativeness of mixing by age	*Φ_1_*	0.75		[63]
Assortativeness of mixing by sexual activity level	*Φ_2_*	0.75		[63]
Degree of preference for partner with 10 years age difference	*Φ_3_*	0.5		[63]
Proportion of adult males in sexual activity levels 1 to 4		0.044, 0.089, 0.195, 0.672		[13]–[15]
Proportion of adult females in sexual activity levels 1 to 4		0.002, 0.026, 0.147, 0.825		[13]–[15]
Ratio of rates of sexual partner acquisition by activity level 1 to 4		100 : 65 : 5 : 1		[13]–[15], [63]
Ratio of rates of sexual partner acquisition by age group 1 to 7		2 : 4 : 6 : 8 : 5: 3 :1		[63]
Average duration of sexual activity		35	year	[13]–[15], [63]
**Demographic**				
Initial population size	*U*	5.7×10^6^	person	[72]
Initial life expectancy for males and females	*1/μ*	49 and 53	year	[72]
Total fertility rate		6.8	births per female	[72]
Sex ratio at birth		1		[63], [72]

* The superscripts represent the disease stage.

Υ Individuals with AIDS were assumed to be sexually inactive during the last 6 months of their life [12], [81], [82].

**Table 2:** Model Parameters for Pre-exposure Chemoprophylaxis (PrEP) Implementation

PARAMETER	SYMBOL‡	UNIT	SENSITIVITY	SCENARIO			REFERENCE
			LHS RANGE (Uniform Distribution)	OPTIMISTIC	NEUTRAL	PESSIMISTIC	
Fraction of individuals enrolled into PrEP (coverage)	*φ*	per year	0.25–0.75	0.75	0.50	0.25	Assumption
Time period to achieve target coverage	*−ln(1−φ)/ν*	year	1–10	1	5	10	Assumption
Effectiveness of PrEP against sensitive virus	*ξθ*		0.25–0.90	0.90	0.60	0.30	Assumption
Effectiveness of PrEP against resistant virus	ξ^R^θ = ι*ξθ		0.00–0.50 * ξθ	0.50 * ξθ	0.25 * ξθ	0.00 * ξθ	Assumption
Fraction of on-PrEP individuals who acquire secondary resistance after infection with sensitive virus (selection)	*π*	per year	0.50–1.0	0.50	0.75	1.00	Assumption
Persistence of primary resistance in individuals who acquire infection while naïve or off PrEP¶ §	*1/ϑ^ΩR′^*	month	1–6	1	3	6	[28], [83]
Persistence of secondary resistance after PrEP discontinuation¶ §	*1/ϑ^ΩR′′^*	month	1–12	1	6	12	[23], [29], [31]
Persistence of primary resistance in individuals who acquire infection while on PrEP after PrEP discontinuation¶ §	*1/ϑ^ΩR′′′^*	month	1–12	1	6	12	[23], [28]–[31], [83]
PrEP permanent discontinuation rate in susceptible individuals†	*σ*	per year	0.00–1.00	0.00	0.05	0.20	Assumption
Infectivity of individuals with primary resistance who acquire infection while naïve or off PrEP	γ^ΩR′^ = ε′*γ^Ω^	per act	0.50–1.00 * γ^Ω^	0.50 * γ^Ω^	0.75 * γ^Ω^	1.00 * γ^Ω^	[35], [36], [84]
Infectivity of individuals with secondary resistance	γ^ΩR′′^ = ε′′*γ^Ω^	per act	0.50–1.00 * γ^Ω^	0.50 * γ^Ω^	0.75 * γ^Ω^	1.00 * γ^Ω^	[23], [24], [34]
Infectivity of individuals with primary resistance who acquire infection while on PrEP	γ^ΩR′′′^ = ε′′′*γ^Ω^	per act	0.50–1.00 * γ^Ω^	0.50 * γ^Ω^	0.75 * γ^Ω^	1.00 * γ^Ω^	[23], [24], [34]–[36], [84]
Probability of transmission of resistant rather than sensitive virus from an individual with primary resistance who acquires infection while naïve or off PrEP	*υ′*		0.50–1.00	0.50	0.75	1.00	[37]–[39]
Probability of transmission of resistant rather than sensitive virus from an individual with secondary resistance	*υ′′*		0.20–1.00	0.20	0.60	1.00	[37]–[39]
Probability of transmission of resistant rather than sensitive virus from an individual with primary resistance who acquires infection while on PrEP	*υ′′′*		0.20–1.00	0.20	0.60	1.00	[37]–[39]
Factor increase in rates of sexual partnership change of individuals, both susceptible and infected, while on PrEP	*r*		1.00–2.00	1.00–2.00	1.00–2.00	1.00–2.00	Assumption

‡ Except for the most relevant, the subscripts/superscripts have been omitted from the symbols for clarity.

¶ For the analyses reported in this paper, we assumed that infections would be detected in the on-PrEP individuals after an average duration of 6 months, when they would stop PrEP and resume baseline sexual activity. Multivariate sensitivity analyses, where we varied the total persistence of drug-resistant virus in infected individuals (i.e. sum of the periods of persistence on- and off-PrEP) between 1 month to 2 years, showed that the model output was not sensitive to changes in this parameter (data not shown).

§ HIV-1 disease progression was assumed the same for drug resistant and drug sensitive virus because: i) a temporary predominance of drug-resistant mutants was assumed in the model; and ii) though lower viremia has been observed in the experimental setting [24], [85], [86], it is unknown whether PrEP would attenuate the course of HIV-1 infection.

† Inverse of the average duration of PrEP use. For the optimistic scenario we assumed the average duration of PrEP use to be equal to the average duration of sexual activity.

### Model Output and Introduction of PrEP

The model's dynamical behavior was investigated using numerical methods. The key model outputs were: HIV-1 prevalence; HIV-1 incidence; cumulative new HIV-1 infections; and cumulative deaths from AIDS. PrEP was introduced (as once daily oral antiretroviral dosing) at endemic equilibrium when HIV-1 prevalence in sexually active adults (15–49 year-olds) was approximately 20%. The implementation of PrEP was simulated both in the absence and presence of sexual disinhibition of the individuals on PrEP, where disinhibition is defined as increased rate of sex partner change. We summed over 20 years of PrEP implementation the number of new infections and the total number of persons on PrEP, to make comparisons between the epidemics with and without PrEP at each simulation time-step. The key output variables employed in these comparisons were: the % change in the cumulative new HIV-1 infections; the ratio of HIV-1 infections prevented to person-years of PrEP; the ratio of HIV-1 infections prevented to persons enrolled in PrEP; and the ratio of the cost of PrEP to the number of infections prevented.

### Effectiveness of PrEP

Our model represents the transmission of HIV-1 as a Poisson process [16]–[18]. The probability of transmission per heterosexual partnership, *β*, between an individual (on PrEP) of gender *g*, activity level *k*, and age *i*, with an (infected) individual of opposite gender *g′*, activity level *l* and age *j* is given by:
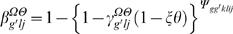
where *Ψ* is the number of sex acts within the partnership; *γ* is the probability of HIV-1 transmission per sex-act (infectivity) based on the disease stage, *Ω*, and drug resistance status, *Θ*, of the infected partner; and *ξθ* is the effectiveness of PrEP. Effectiveness is defined as the probability of preventing HIV-1 transmission per sex-act through PrEP and is given by the product of the average efficacy of PrEP, *ξ* (the degree of protection provided, from HIV-1 transmission per sex-act) and the average level of adherence to PrEP, *θ* (assuming once daily dosing and that doses are missed at random). In a partnership, where the infected partner harbors major drug-resistant variants (discussed below), the probability of transmission of resistant virus is *υβ*, while that of wild-type virus is *(1−υ)β*, and the effectiveness of PrEP against resistant virus is *ιξθ*. The parameters *ξ, θ, υ* and *ι* assume values between 0 and 1 (Table 2).

### Modeling Drug Resistance

We sub-classified the HIV-1 infected individuals based on their PrEP status (naïve, on or off), type of drug resistance (primary or secondary), and simplified population dynamics of drug-resistant HIV-1 (persistence or reversion), to represent the individuals' drug resistance status (Figure 1 and Table 2). Our model assumptions for HIV-1 drug resistance are as follows. In an infected individual, the HIV-1 population is comprised of a set of related variants, termed as viral quasispecies [19]. Before the introduction of PrEP, all HIV-1 infected individuals harbor a dominant population of wild-type (drug-sensitive) virus [20]. Drug-resistant mutants are selected by drug pressure in a fraction (termed selection, *π*, having a value between 50% to 100%) of those individuals who become infected by wild-type virus while on PrEP (e.g. emergence of mutants with: K65R with tenofovir [21]–[23]; M184V with emtricitabine [24]; and M184V+K65R with tenofovir+emtricitabine [25], [26]). This type of resistance is termed secondary resistance [27]. A proportion of susceptible individuals, both on and not on PrEP, become infected by drug-resistant mutants through transmission from their sexual partner. This type of resistance is termed primary resistance [27]. Upon removal of selection pressure, either by discontinuation of PrEP [23] or transmission to a drug naïve individual [28], the drug-resistant mutants decline after a period of persistence, due to outgrowth by wild-type virus (reversion) [29]–[31]. Prior to reversion, the drug-resistant mutants are the dominant (major) viral variants [20], [32], whereas following reversion these become minor variants [32], [33]. Compared to individuals with wild-type virus, individuals with drug-resistant variants can have: i) decreased probability of transmission per sex act (infectivity, *γ^ΩΘ^*, having a relative value of 50% to 100%) due to lower level viremia from PrEP use [23], [24] or from diminished viral replicative fitness [34]–[36]; and ii) decreased viral transmission fitness (probability per partnership that a resistant rather than wild-type virus will be transmitted, *υ*, with a value between 20% to 100%) [37]–[39]; but the same rate of disease progression due to temporary predominance of drug-resistant mutants. Individuals with minor drug-resistant variants behave as individuals with wild-type virus in terms of infectivity and disease progression and likewise do not transmit drug resistant mutants. The re-emergence of major variants due to subsequent drug challenge (e.g. antiretroviral therapy) [40], [41] was not modeled.

### Sensitivity Analyses

We performed sensitivity analyses [42] to determine the relative influence of PrEP-related model input parameters (Table 2) on the predicted decrease in new infections. For multivariate time-dependent sensitivity analyses, we rank transformed input and output data obtained using Latin hypercube sampling [43], [44] and 1000 simulation runs for epidemic scenarios with and without sexual disinhibition, and from this derived partial rank correlation coefficients (PRCCs) and standardized rank regression coefficients (SRRCs) [45], [46].

### PrEP Scenarios

The impact of PrEP was determined by simulating three different scenarios: namely, optimistic, neutral and pessimistic (Table 2). For each of these scenarios, we simulated: i) the implementation of PrEP in the sexually active population in general (non-targeted strategy); ii) PrEP targeted to the two highest sexual activity levels (targeted-by-activity strategy); and iii) PrEP initiation targeted to the group 15–20 years of age (targeted-by-age strategy). We performed univariate sensitivity analysis for each scenario, in which we measured the change in infections arising from variation of each PrEP-related input parameter over its specified range (Table 2). We also analyzed the interplay between key PrEP-related input parameters and the number of infections prevented. Finally, we estimated the potential reduction in the number of new adult infections in all of southern sub-Saharan Africa, by extrapolating our results to current epidemiological data from that region [47].

*Software:* Model construction, simulations and sensitivity runs were implemented concurrently in Berkeley Madonna (version 8.3.8; Robert I. Macey and George F. Oster) and Vensim DSS (version 5.5d; Ventana Systems, Inc.). Data were analyzed using Microsoft Excel (version 11.8; Microsoft Corporation) and Stata SE (version 9.2; StataCorp LP).

## Results

Our mathematical model stratifies the population based on gender, age, sexual activity level, disease state, PrEP status, and HIV-1 drug resistance (Figure 1), and its dynamical behavior is analyzed numerically. We introduced PrEP at endemic equilibrium and simulated optimistic, neutral and pessimistic scenarios (Table 2). For each scenario we simulated three strategies of PrEP implementation: i) in the sexually active population in general (non-targeted strategy); ii) in the two highest sexual activity levels (targeted-by-activity strategy); and iii) in the group15–20 years of age (targeted-by-age strategy). Each strategy was simulated both with and without sexual disinhibition of the individuals on PrEP. To determine the epidemiological impact of PrEP, we compared the epidemics with and without PrEP up to 20 years and determined the % change in the cumulative new HIV-1 infections; the ratio of HIV-1 infections averted to person-years of PrEP; the ratio of HIV-1 infections averted to persons enrolled in PrEP; and the ratio of the cost of person-years of PrEP to the number of infections averted.

**Figure 2:**
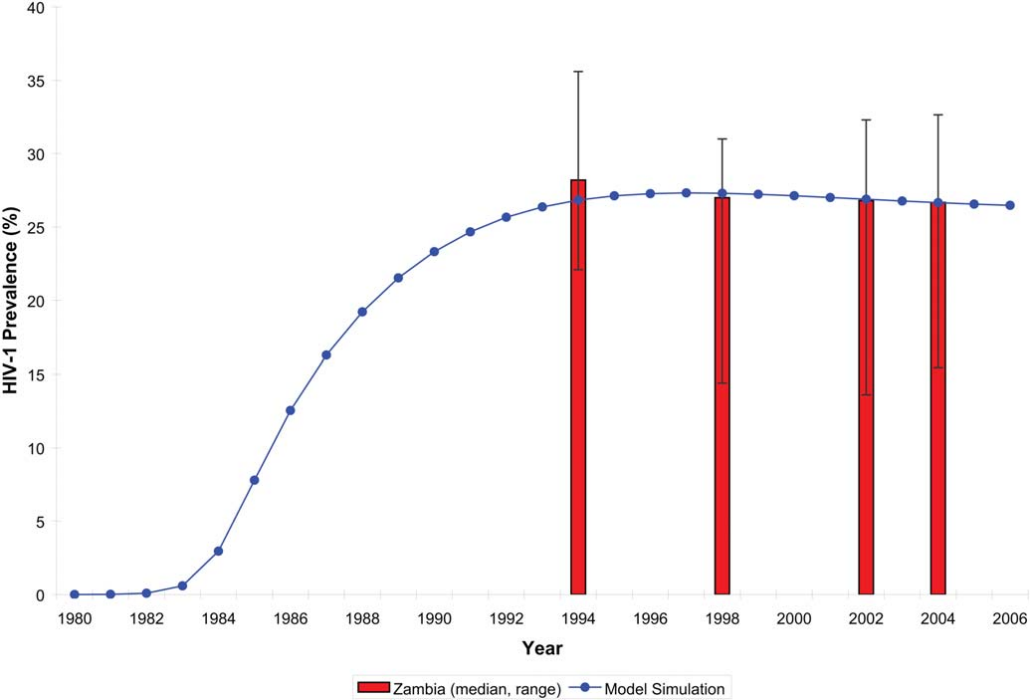
Trends in HIV-1 Prevalence among Urban Antenatal Clinic Attendees in Zambia from 1994 to 2004 [49] and the Simulated Adult Female Population.

In the simulated epidemic, adult HIV-1 prevalence was 20% at endemic equilibrium with the ratio of female to male prevalence of 1.66 [48]. Simulated trends in female prevalence are shown in Figure 2. They mimic observed patterns among urban antenatal clinic attendees in Zambia [49].

The results for both univariate and multivariate sensitivity analyses of the predicted impact of PrEP were similar, thus only multivariate results are presented. Table 3 shows sensitivity analyses of the predicted decline in cumulative new HIV-1 infections for 20 years after PrEP implementation. In general, coefficient (PRCC and SRCC) values near 1 indicate a strong positive influence of the input variable on prevention of infections, whereas values near -1 indicate a strong negative influence. Values near 0 indicate little, if any, influence [50]. In the scenario without sexual disinhibition occurring, the rate of PrEP discontinuation (inverse of the average duration of PrEP use) was the strongest determinant overall of reduction in infections and its effect persisted over time (PRCC ranged from −0.94 at year 5 to −0.97 at year 20). The next most important determinants were the effectiveness of PrEP (composite of efficacy and adherence) and the fraction of individuals covered (coverage) by PrEP (PRCCs of 0.92 and 0.88 at year 10, respectively), with both parameters having a positive influence. The time to achieve target coverage had a strong negative influence on infections prevented at year 5 (PRCC of −0.82), which declined over long durations of time. A weak negative influence (PRCC of −0.13) was seen for secondary drug resistance (resistance developing on PrEP), though this persisted over time.

**Table 3:** Results of Sensitivity Analyses for Model Parameters Affecting the Decrease in Cumulative New HIV-1 Infections (%)

PARAMETER	NO SEXUAL DISINHIBITION				SEXUAL DISINHIBITION			
	Year 5	Year 10	Year 15	Year 20	Year 5	Year 10	Year 15	Year 20
	***PARTIAL RANK CORRELATION COEFFICIENTS*** *							
Effectiveness of PrEP against sensitive virus	0.94	0.92	0.91	0.89	0.91	0.89	0.88	0.87
Fraction of individuals enrolled into PrEP	0.92	0.88	0.86	0.84	0.54	0.45	0.41	0.40
Probability of transmission of resistant virus from *Y^R′′^* ^ж ^ ‡					−0.07	−0.08	−0.08	−0.07
Infectivity of *Y^R′′^* ‡	−0.13	−0.13	−0.14	−0.14	−0.16	−0.16	−0.16	−0.17
Time period to achieve target coverage	−0.82	−0.13			−0.36			
PrEP permanent discontinuation rate	−0.94	−0.96	−0.97	−0.97	−0.58	−0.66	−0.67	−0.67
Increase in sexual risk behavior					−0.77	−0.75	−0.74	−0.73
	***STANDARDIZED RANK REGRESSION COEFFICIENTS*** *							
Effectiveness of PrEP against sensitive virus	0.56	0.50	0.46	0.44	0.76	0.74	0.72	0.71
Fraction of individuals enrolled into PrEP	0.49	0.39	0.35	0.34	0.22	0.19	0.18	0.17
Probability of transmission of resistant virus from *Y^R′′^* ^ж ^ ‡					−0.02	−0.03	−0.03	−0.03
Infectivity of *Y^R′′^* ‡	−0.03	−0.03	−0.03	−0.03	−0.06	−0.06	−0.06	−0.07
Time period to achieve target coverage	−0.30	−0.03			−0.13			
PrEP permanent discontinuation rate	−0.56	−0.75	−0.79	−0.81	−0.24	−0.32	−0.35	−0.36
Increase in sexual risk behavior					−0.42	−0.42	−0.42	−0.43

* The p-value is 0.0000 except for ж where the p-value < 0.05.

‡ *Y^R′′^* represents individuals with secondary resistance.

In the scenario in which sexual disinhibition occurred among individuals on PrEP, the effectiveness of PrEP emerged as the strongest positive determinant of infections prevented with a PRCC ranging from 0.87 to 0.91. The increase in at-risk behavior was the strongest negative determinant of infections prevented having a PRCC of −0.75 at year 10. Though PrEP discontinuation rate and coverage remained significant with disinhibition scenario, their effect was attenuated on average by about 33% and 50%, respectively, compared to the scenario without disinhibition.

SRRC values confirmed the above findings. In the absence of sexual disinhibition, the influence on infections prevented was strongest for the rate of PrEP discontinuation (SRRC range: −0.56 to −0.81) followed by effectiveness (SRRC range: 0.44 to 0.56) and coverage (SRRC range: 0.34 to 0.49). With sexual disinhibition (SRCC of −0.42), the effectiveness of PrEP became the predominant influence on the infections prevented (SRRC range: 0.71 to 0.76).

Table 4 compares the outcomes in the optimistic, neutral and pessimistic scenarios 10 years after the introduction of PrEP. These scenarios respectively assume optimistic, neutral and pessimistic sets of PrEP-related input parameters in Table 2. The potential impact of PrEP was impressive for the optimistic scenario, but was negligible for the pessimistic scenario, illustrating the importance of key chemoprophylaxis parameters on outcome. For each scenario, the greatest decline in infections was achieved with the non-targeted strategy, whereas the lowest cost of PrEP per infection averted was obtained with the targeted-by-activity strategy. Specifically, a 74% reduction in infections occurred for the optimistic scenario, 24.9% for the neutral scenario and 3.3% for the pessimistic scenario with the non-targeted strategy. These figures declined to 28.8%, 6.8% and 0.8%, respectively, with the targeted-by-activity strategy. However, the cost of person-years of PrEP per infection averted over the 10 year intervention time span fell substantially with the targeted strategy; from $6,812 to $638 for the optimistic scenario, from $9,629 to $ 1,160 for the neutral scenario, and from $20,164 to $2,949 for the pessimistic scenario. The targeted-by age strategy yielded intermediate declines in infections (45.5%, 14.5% and 2.0%), although the cost of person-years of PrEP per infection averted remained high ($5,723, $8,968 and $20,202). Overall, the numbers of infections averted per person-year of PrEP and per person enrolled in PrEP were highest for the optimistic targeted-by-activity strategy (0.33 and 1.74). Similar results were seen after 20 years of PrEP (data not shown).

**Table 4:** Outcomes for Optimistic, Neutral and Pessimistic Scenarios after Ten Years of PrEP Implementation

Outcome	Non-Targeted			Targeted by Sexual Activity			Targeted by Age Group		
	Optimistic	Neutral	Pessimistic	Optimistic	Neutral	Pessimistic	Optimistic	Neutral	Pessimistic
Decline in Cumulative New HIV-1 Infections (%)	74.0	24.9	3.3	28.8	6.8	0.8	45.5	14.5	2.0
Decline in Cumulative New HIV-1 Infections (%); *r = *2‡	62.7	1.3	−7.0	17.7	−1.9	−2.5	36.5	0.1	−4.4
Infections Averted per Person-Year of PrEP	0.03	0.02	0.01	0.33	0.18	0.07	0.04	0.02	0.01
Infections Averted per Person Enrolled in PrEP	0.21	0.11	0.03	1.74	0.62	0.15	0.21	0.10	0.03
Cost of Person-Years of PrEP per Infection Averted($)¶	22,918	32,398	67,842	2,147	3,904	9,923	19,254	30,173	67,970
Cost of Person-Years of PrEP per Infection Averted($)§	10,397	14,697	30,776	974	1,771	4,502	8,734	13,688	30,834
Cost of Person-Years of PrEP per Infection Averted($)†	6,812	9,629	20,164	638	1,160	2,949	5,723	8,968	20,202

‡ Assuming a 100% increase in at-risk behavior.

¶ Assuming $700 per person-year of PrEP; the market price of a generic version of tenofovir (Tenvir) manufactured by Cipla in India [87].

§ Assuming $318 per person-year of PrEP ($0.87/day); the current cost of manufacturing tenofovir+emtricitabine (Truvada) by Gilead [88].

† Assuming $208 per person-year of PrEP ($0.57/day); the current cost of manufacturing tenofovir (Viread) by Gilead [88].

Costs (defined as drug costs per person-year of PrEP) and health benefits (infections averted) are presented in their undiscounted form for clarity [80]. Costs of PrEP exclude all other costs e.g. drug distribution, pharmacy and clinical services, communications and education, laboratory, treatment of complications including resistance, and counseling. Analyses also exclude the consequences of HIV-1 infection including costs of provision of antiretroviral therapy.

**Figure 3:**
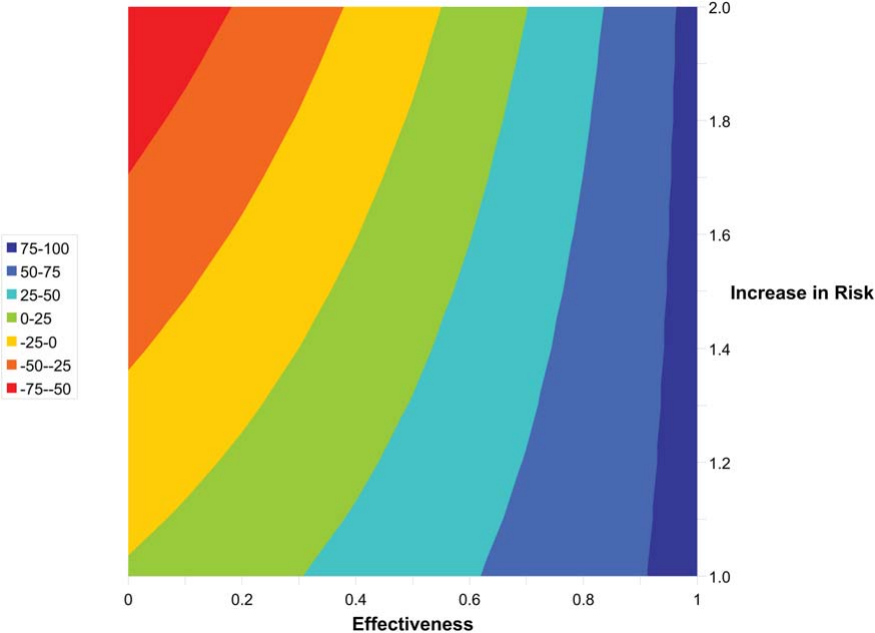
Contour Graph for Decline in Cumulative Infections (%) as a Function of Effectiveness of PrEP and Increase in Risk Behavior Assuming Optimistic Scenario. Negative numbers reflect increase in infections.

Sexual disinhibition of individuals on PrEP progressively eroded the declines in infections for all scenarios (Table 4), although this effect was modest for the optimistic scenario. Specifically, at year 10 in the optimistic scenario with a 100% increase in at-risk behavior, the decline in infections was 62.7% for the non-targeted strategy and 17.7% for the targeted-by activity strategy (reduced from 74% and 28.8%, respectively, without disinhibition). The infections increased by 1.9% for the neutral scenario with targeted-by activity strategy, and increased by 7%, 2.5% and 4.4% for the three pessimistic scenario strategies (non-targeted, targeted by sexual activity and targeted by age group). Such increases were also seen with the optimistic scenario when lower levels of effectiveness were assumed. For example, at 50% effectiveness, infections increased by 8% for the non-targeted strategy as the result of a 100% increase in at-risk behavior (Figure 3). With sexual disinhibition, the decline in infections was also influenced negatively by the infectivity of individuals with secondary resistance and the probability of transmission of resistant virus from individuals with secondary resistance; however, these effects were weak with PRCCs of −0.16 and −0.08, respectively, at year 10 (Table 3). Other input parameters related to drug-resistance (Table 2) were not significantly associated with outcome.

Table 5 quantifies the predicted impact of PrEP for selected countries in southern Sub-Saharan Africa, as well as for the overall region, where the median country-prevalence of HIV-1 is about 20% [47]. In South Africa, up to 1.5 million new HIV-1 infections could be averted over 10 years by PrEP coverage of 75% of high sexual activity groups [51]. The corresponding estimate of the infections averted for Zambia is 0.36 million, for Botswana 0.13 million and for Lesotho 0.09 million. For southern sub-Saharan Africa as a whole up to 3.2 million new HIV-1 infections could be averted over 10 years at a cost of roughly $2.0 billion for PrEP. All these estimates assume high levels of efficacy and adherence to PrEP.

**Table 5:** Potential Impact of PrEP Introduced in 2007 on HIV-1 Infections in Southern Sub-Saharan Africa‡ ¶

Region/Country	Baseline Adult HIV Prevalence %	Baseline Adult HIV Incidence %	Baseline Adult Population people	Population Growth Rate %	Cumulative New HIV Infections Averted after 10 Years	
					Optimistic Scenario & Targeted by Sexual Activity	
					No Disinhibition	100% Disinhibition
Lesotho	23.2	4.8	865 000	0.1	92 710	56 942
Botswana	24.1	6.7	909 000	0.1	132 870	81 608
Zambia	17.0	2.6	5 281 000	1.7	361 132	221 803
South Africa	18.8	2.4	25 204 000	0.8	1 477 691	907 581
Southern Sub-Saharan Africa§	19.6†		54 886 000		2 713 746–3 166 037	1 666 752–1 944 544

‡ These are conservative projections based on estimates of the size of adult population [47], [71] and assuming constant incidence [73]–[75], [89], prevalence [47] and growth rate [47].

¶ For southern sub-Saharan Africa overall, projections are based on the UNAIDS/WHO statement that the total number of infections in this region were 1.1 million for three consecutive years including 2005 [75]. With this estimate as a constant, low projection assumes 86% of these infections occur in adults, while the high projection assumes the full estimate.

§ Excludes Angola, Madagascar, Mauritius and Seychelles.

† Refers to median country-prevalence.

## Discussion

Data from animal studies show that systemic antiretrovirals can prevent infection of macaques by simian immunodeficiency virus [1], [3]–[5]. The safety and efficacy of once daily oral antiretroviral PrEP in humans are under clinical trials in the Unites Sates, Latin America, Africa and Asia [7], [8]. However, these studies are not designed to address the population-level impact of PrEP on rates of HIV-1 transmission over many years. Using a carefully stratified and well-parameterized deterministic model of HIV-1 transmission, our analyses suggest that PrEP could have a profound impact on the HIV-1 epidemic, if effectiveness is high (high levels of efficacy and adherence) and usage persists over a decade or more; that is over much of the typical duration of sexual activity of an individual. Though the maximum effect of PrEP was observed at the highest level of coverage (75% of susceptible sexually active individuals) with good continuous adherence, such coverage and adherence are not realistic. Furthermore, the cost of untargeted PrEP per infection prevented is relatively high at $6,812. PrEP targeted to persons with greatest sexual activity produced significant declines in infections and had the lowest cost of person-years of PrEP per infection averted at $638. This result is noteworthy for two reasons: the highest activity groups comprised only 13.3% and 2.8% of the male and female model populations; and PrEP was introduced at endemic equilibrium when these high activity groups become saturated and play a lesser role in the spread of HIV-1 compared to earlier stage epidemics [52]. Models of HIV-1 vaccine implementation have also suggested that targeting by sexual activity could have a significant epidemiological impact [53]. In contrast to these vaccine models, we found that targeting by age group had less of an impact than a non-targeted approach and very similar estimates of cost of person-years of PrEP per infection averted. This is because, unlike the assumption of a one time (± booster) vaccination, PrEP requires continual use in the presence of ongoing risk of sexual transmission.

Sensitivity analyses showed that the effectiveness of PrEP was the most important determinant of the magnitude of decline in infections. This is especially the case in the scenario with sexual disinhibition of the individuals on PrEP. The high effectiveness assumed in the optimistic scenario was also the foremost reason why this scenario yielded the best outcomes overall, including cost of person-years of PrEP per infection averted. When effectiveness was lower, infections increased for all scenarios with increased risk taking behaviors of those on PrEP. The decline in infections was also very sensitive to the PrEP discontinuation rate (inverse of the average duration of PrEP use) and the level of coverage. We modeled the PrEP discontinuation rate as distinct from adherence, which was represented within our composite parameter of effectiveness. Our data suggest that continual access to PrEP would be of great importance and permanent discontinuation would undermine the epidemiological gains if PrEP use was short-lived in relation to individuals' typical duration of sexual activity.

Our representation of the evolution and transmission of drug resistance is crude. Further model development is required in this area and will be the subject of additional study. Nevertheless, the parameters directly related to drug resistance did not emerge as key determinants of the outcome of PrEP. These results may be explained by a greater contribution of other parameters impacting HIV-1 transmission, such as PrEP effectiveness.

The feasibility of PrEP as an HIV-1 prevention strategy would not only depend on its safety and efficacy, but also on its incremental cost-effectiveness compared to other intervention strategies in resource-poor settings. Our simple comparison between implementation of PrEP to a “do nothing” strategy revealed that the cost of person-years of PrEP per infection averted for the optimistic scenario with targeted-by-activity strategy of $638–$2147, compared favorably with the projected cost of $3900 per infection averted over the period 2005–2015 with the UNAIDS comprehensive prevention package [54]. This same study projected a savings of $4700 in forgone treatment and care costs. Using mathematical models, other investigators have reported that PrEP is a cost-effective strategy among high-risk men who have sex with men in New York City [55], and among populations in low-income settings [56].

Sub-Saharan Africa has about 63% of the HIV-infected population of the world totaling 22.4 million adults [47], [57]. Though epidemics of various characteristics are affecting this region [58], the vast majority of the population are not yet infected with HIV-1 and thus effective prevention strategies are urgently needed. Our analyses indicate that approximately 2.7 to 3.2 million new HIV-1 infections could be averted in southern sub-Saharan Africa over the next 10 years by targeting PrEP to population groups with the highest sexual activity concomitant with preventing increased risk behaviors in those on PrEP. It is not always easy, however, to identify those with high sexual activity patterns, except if they are involved in commercial sexual activities [14], [59].

The estimate of the number of infections averted for South Africa alone is 1.5 million infections. This high individual and public health benefit will require sustained access to PrEP. In addition, the integration of PrEP programs with voluntary counseling and testing services and other prevention programs (e.g. promotion of condom use, circumcision, and identification and treatment of sexually transmitted diseases (STDs) will be key in controlling spread of HIV-1) [60].

There are some important limitations of our current model structure and the assumptions embedded within it. The precise quantitative detail of our predictions will be affected by variations in the structure and sexual activity patterns of different populations, for which data are very limited, especially on sexual mixing patterns. However, we employed a well-established template of sexual behavior [61]–[65], with robust epidemiological and demographic parameterization, broadly applicable to southern sub-Saharan Africa. The actual impact of PrEP will depend on the PrEP agent or agents used as well as the physiological, behavioral and viral characteristics of the HIV-1 infected target population. Primate studies of PrEP suggest superiority of tenofovir plus emtricitabine over tenofovir alone [1], [2]. Natural polymorphisms in HIV-1 subtypes have been postulated to play an important role in drug resistance pathways [66], including the propensity of HIV-1 subtype C virus that is predominant in Sub-Saharan Africa [67], for more frequent and rapid development of the K65R tenofovir-resistance mutation, noted by some investigators [68], [69] though not by others [70]. Although there is substantial uncertainty regarding PrEP-related parameters, we employed wide ranges (within plausible bounds) for our input parameters and performed extensive sensitivity analyses. There are significant differences between the demographic and HIV/AIDS epidemiological trends estimates predicted by different agencies, largely as a result of the methods employed in analysis and prediction [15], [47], [71], [72]. In addition, except for South Africa [73], estimates of HIV-1 incidence have not been measured directly at the population level in most African countries, and reliable country-specific estimates are rarely available excepting from a few well-defined study sites with long term surveillance [74]. We elected to employ the demographic and HIV/AIDS epidemiological estimates from UNAIDS where applicable [47], [71], [75]. Our optimistic analyses assume a high level of effectiveness for PrEP, which may not be the case because of more limited drug activity and/or medication adherence. However, data on both efficacy of the potential PrEP agents [1], and adherence in Africa [76] justify some degree of optimism. In a macaque study [1] in which animals received weekly rectal simian human immunodeficiency virus challenges, 83% (5/6) of the controls became infected after 14 challenges, whereas 100% (6/6) of the macaques that received subcutaneously a combination of 22 mg tenofovir and 20 mg emtricitabine per kg once daily remained uninfected . A meta-analysis [76] of 31 North American studies (17,573 patients total) indicated a pooled estimate of 55% of the populations achieving adequate levels of adherence, whereas analysis of 27 African studies (12,116 patients total) indicated a pooled estimate of 77%. About 71% of the former and 66% of the latter studies used patient self-report to assess adherence and similar thresholds for adherence monitoring (>80% to 100%). The authors concluded that although adherence remained a concern in North America, favorable levels of adherence could be achieved in sub-Saharan Africa.

We excluded from our analyses the impact of antiretroviral therapy for infected persons [9], various other influences on transmission (e.g. STDs [77], [78], circumcision [79] and condom use [16]), as well as a formal cost-effectiveness analysis [80]. These and other refinements will be addressed in future work. Nevertheless, the key conclusion of this study is that PrEP can be a cost effective intervention given high efficacy, good adherence and long-term use, especially if sexual disinhibition is prevented.

## Supporting Information

Appendix S1 (0.16 MB DOC)
