# Supplementary material for: Potential Impact of Antiretroviral Chemoprophylaxis on HIV-1 Transmission in Resource-Limited Settings
Source: PLoS One. 2007 Sep 19;2(9):e875. doi: 10.1371/journal.pone.0000875 (PMC1975470; doi:10.1371/journal.pone.0000875)
Supplement: Appendix S1 — (0.16 MB DOC) [file pone.0000875.s001.doc]

**Appendix S1**

Using a differential equation framework, our model represents the transmission dynamics of HIV-1 infection, disease progression and implementation of PrEP in a heterosexual population, which is stratified into groups based on gender, age, sexual activity, disease state, PrEP status and HIV-1 drug resistance.

Individuals can either be susceptible *(Χ)* to or infected with HIV-1 *(Υ)*. After acquisition of HIV-1, individuals pass through three successive stages *(Ω)* of infection followed by death from AIDS. The stages include: i) recent infection *(~~Ω~~ for Ω=1)*; ii) chronic infection; and iii) AIDS (CD4 cells < 200/mm^3^). Each stage is characterized by a stage-transition rate (disease progression; *ω*) and a probability of transmission per sex act (infectivity; *γ*). In an epidemic with implementation of PrEP, the susceptible individuals are categorized as PrEP-naïve *(Χ^N^)*, on PrEP *(Χ^Ρ^)* and off PrEP *(Χ^O^)*. Susceptible individuals continue using PrEP with imperfect adherence *(θ)* or permanently abandon PrEP at a discontinuation rate *(σ)*. Individuals who are PrEP-naïve, or off of PrEP, can become HIV-1 infected, due to either wild-type (drug-sensitive) virus *(Y^S^)* or drug-resistant virus *(Y^R'^)*. Individuals on PrEP can also become HIV-1 infected due to drug-resistant virus *(Y^PR'''^)* or due to wild-type virus. A fraction *(π)* of the latter develop drug resistance due to selective drug pressure *(Y^PR''^)* while the rest remain drug-sensitive *(Y^PS^)*. Individuals on PrEP after becoming infected stop using PrEP *(Y^OS^, Y^OR''^ and Y^OR'''^)* at a stop rate *(η)*. Subsequently, individuals with predominantly drug-resistant virus, transition into individuals with predominantly wild-type virus *(Y^S'^, Y^OS''^and Y^OS'''^)*, at a reversion rate *(ϑ)*.

The model represents two strategies of PrEP implementation [1]. In the cohort-based strategy, a fraction *(φ)* of new entrants into the sexually active population adopt PrEP. In the population-based strategy, a proportion of the sexually active population take up PrEP over a given time period based on a defined rate *(ν)*. PrEP implementation occurs in two phases. The induction phase extends from the time of introduction of PrEP to the time when target coverage (the desired fraction of the population to be enrolled into the PrEP program) is achieved. This is followed by the maintenance phase where target coverage is continued. During the induction phase, the model uses a combined cohort-based and population-based PrEP implementation strategy. Only the cohort-based strategy is continued through the maintenance phase. PrEP is introduced at the endemic prevalence and three different scenarios are simulated: optimistic, neutral and pessimistic, alternatively with and without sexual disinhibition. For each scenario, three different strategies are examined: i) the implementation of PrEP in the sexually active population in general (non-targeted strategy); ii) PrEP targeted to the two highest sexual activity levels (targeted-by-activity strategy); and iii) PrEP initiation targeted to the group 15-20 years of age (targeted-by-age strategy).

Both males and females are represented from birth until death and are divided into distinct five-year age groups (except for 0-1 and 1-4 year age groups) with group-specific aging *(α)* and mortality *(μ)* rates (and female fertility rates). However, the focus of our study is the sexually active population aged 15-49 years. Individuals are assigned one of four possible levels of sexual activity (very high, high, medium, and low) and their heterosexual interaction is defined both by a hybrid (random and preferred) sexual mixing pattern *(ρ)* and a dynamic rate of sexual partnership change *(ĉ)*, as described by Garnett and Anderson [2,3]. The model represents sexual disinhibition as an increase *(r)* in rates of sexual partnership change (at-risk behavior) of individuals, both susceptible and infected, while on PrEP [1].

The following set of differential equations describes the change *(∂)* in populations of susceptible *(X)* and infected *(Y)* individuals with respect to change in time *(∂τ)*. The subscripts represent gender *(g)*, sexual activity class *(k)* and age group *(i)*. The superscripts represent PrEP status *(N, P or O)*, disease stage *(Ω)* and drug-resistance status *(S, S′, S′′, S′′′, R′, R′′, or R′′′).*

$\frac{\partial X_{gki}^{N}}{\partial\tau}=\left(1-\varphi_{gki}\right) \alpha_{gki-1} X_{gki-1}^{N} - \left(\nu_{gki}+\mu_{gki}+\alpha_{gki}+\lambda_{gki}^{S}+\lambda_{gki}^{R}\right) X_{gki}^{N}$ (1)

$\frac{\partial X_{gki}^{O}}{\partial\tau}=\alpha_{gki-1} X_{gki-1}^{O} + \sigma_{gki }X_{gki}^{P} - \left(\mu_{gki}+\alpha_{gki}+\lambda_{gki}^{S}+\lambda_{gki}^{R}\right) X_{gki}^{O}$ (2)

$\frac{\partial X_{gki}^{P}}{\partial\tau}=\left(\varphi_{gki}\right) \alpha_{gki-1} X_{gki-1}^{N} + \alpha_{gki-1} X_{gki-1}^{P} +\nu_{gki} X_{gki}^{N} - \left(\sigma_{gki}+\mu_{gki}+\alpha_{gki}+\lambda_{gki}^{S}+\lambda_{gki}^{R}\right) X_{gki}^{P}$ (3)

(4)

$\frac{\partial Y_{gki}^{\Omega NS}}{\partial\tau}=\omega_{gki}^{\Omega-1S} Y_{gki}^{\Omega-1NS} + \alpha_{gki-1} Y_{gki-1}^{\Omega NS} - \left(\omega_{gki}^{\Omega S}+\mu_{gki}+\alpha_{gki}\right) Y_{gki}^{\Omega NS}$ (5)

(6)

(7)

(8)

(9)

(10)

$\frac{\partial Y_{gki}^{\Omega PS}}{\partial\tau}=\omega_{gki}^{\Omega-1S} Y_{gki}^{\Omega-1PS} + \alpha_{gki-1} Y_{gki-1}^{\Omega PS} - \left(\eta_{gki}^{\Omega S}+\omega_{gki}^{\Omega S}+\mu_{gki}+\alpha_{gki}\right) Y_{gki}^{\Omega PS}$ (11)

(12)

$\frac{\partial Y_{gki}^{\Omega OS}}{\partial\tau}=\eta_{gki}^{\Omega S} Y_{gki}^{\Omega PS} + \omega_{gki}^{\Omega-1S} Y_{gki}^{\Omega-1OS}+ \alpha_{gki-1} Y_{gki-1}^{\Omega OS} - \left(\omega_{gki}^{\Omega S}+\mu_{gki}+\alpha_{gki}\right) Y_{gki}^{\Omega OS}$ (13)

(14)

(15)

(16)

(17)

(18)

(19)

(20)

(21)

(22)

(23)

(24)

(25)

The per capita force of infection for drug-sensitive *(λ^S^)* and drug-resistant *(λ^R^)* HIV-1 is given by:

(26)

(27)

where $\hat{c}_{gklij}$ is the rate at which an individual of gender *g*, activity level *k* and age *i* changes partners belonging to the opposite gender *g′*, activity level *l* and age *j*. The probability of formation of such a partnership is given by$p_{gklij}$. The term is the total number of persons of opposite gender *g′*, activity level *l* and age *j*, while is the number of infected persons among these, having disease stage *Ω* , PrEP status ** (* = N, P or O)* and drug-resistance status *Θ (Θ = S, S′, S′′, S′′′, R′, R′′, or R′′′ )*. The probability that an individual (with a specific drug-resistance status) with drug-resistant virus will transmit resistant virus is given by *υ.*

The term is the per partnership probability of transmission of HIV-1 to an individual of gender *g* from a partner of opposite gender *g′*, activity level *l*, age *j*, disease stage *Ω* and drug-resistance status *Θ* and is given by the formula:

(28)

where denotes the probability of transmission per sex act and is the total number of sex acts per partnership between an individual of gender *g*, activity level *k* and age *i* and an individual of opposite gender *g′*, activity level *l* and age *j*. The effectiveness of PrEP is represented by *ξθ*, and is defined as the probability of preventing HIV-1 transmission per sex-act through PrEP. It is the product of the average degree of protection from HIV-1 transmission per sex-act provided by PrEP, *ξ* (efficacy), and the average degree of adherence, *θ*, to PrEP (assuming once daily dosing and that doses are missed at random) [4].

The sexual mixing matrices are defined as [2]:

(29)

(30)

(31)

where *Φ_1_* is the extent of assortative mixing between age groups and *Φ_2_* is the extent of assortative mixing between sexual activity levels (*Φ* = 0 for entirely assortative mixing and *Φ* = 1 for entirely random mixing). The term *δ_ij_* is the Kronecker delta (*δ_ij_* = 1 if i = j and *δ_kl_* = 1 if k = l, 0 otherwise). For males, *Φ_3_* is the degree to which males prefer partnerships with females ten years younger over females of their own age group (as illustrated above), while for females *Φ_3_* is the degree to which females prefer males ten years older over males of their own age group.

The balance between the supply and demand of sexual partnerships is achieved by applying the rule [3]:

(32)

In addition, continual adjustment is made for the imbalance arising from the differential AIDS mortality in the sexual activity classes using the following rules [3]:

(33)

(34)

$\hat{c}_{gki}=c_{gki}\Delta_{klij}^{\phi}$ (35)

where $\Delta_{klij}$is the level of imbalance and *ϕ* is the amount by which one gender compensates. When *ϕ* = 0.5, both genders compensate equally (our model’s assumption).

**References**

1. Anderson R, Hanson M (2005) Potential public health impact of imperfect HIV type 1 vaccines. J Infect Dis 191(Suppl 1): 85-96.

2. Garnett GP, Anderson RM (1993) Factors controlling the spread of HIV in heterosexual communities in developing countries: patterns of mixing between different age and sexual activity classes. Philos Trans R Soc Lond B Biol Sci 342: 137-159.

3. Garnett GP, Anderson RM (1994) Balancing sexual partnerships in an age and activity stratified model of HIV transmission in heterosexual populations. IMA J Math Appl Med Biol 11: 161-192.

4. Weinstein MC, Graham J, Siegel JE, Fineberg HV (1989) Cost-effectiveness analysis of AIDS prevention programs: concepts, complications and illustrations. In: Turner C, Miller H, Moses L, editors. Confronting AIDS: Sexual behavior and intravenous drug use. Washington DC: National Academy Press. pp. 471-499.
